# Green Fluorescent Protein-based Expression Screening of Membrane Proteins in *Escherichia coli*

**DOI:** 10.3791/52357

**Published:** 2015-01-06

**Authors:** Louise E. Bird, Heather Rada, Anil Verma, Raphael Gasper, James Birch, Matthew Jennions, Jan Lӧwe, Isabel Moraes, Raymond J. Owens

**Affiliations:** ^1^Oxford Protein Production Facility, Research Complex at Harwell; ^2^Protein Crystallography Group, Ruhr University; ^3^MRC Laboratory of Molecular Biology, Cambridge Biomedical Campus; ^4^Membrane Protein Laboratory, Diamond Light Source

**Keywords:** Microbiology, Issue 95, membrane proteins, green fluorescent protein, fluorescence detection, *Escherichia coli*, expression screening

## Abstract

The production of recombinant membrane proteins for structural and functional studies remains technically challenging due to low levels of expression and the inherent instability of many membrane proteins once solubilized in detergents. A protocol is described that combines ligation independent cloning of membrane proteins as GFP fusions with expression in *Escherichia coli *detected by GFP fluorescence. This enables the construction and expression screening of multiple membrane protein/variants to identify candidates suitable for further investment of time and effort. The GFP reporter is used in a primary screen of expression by visualizing GFP fluorescence following SDS polyacrylamide gel electrophoresis (SDS-PAGE). Membrane proteins that show both a high expression level with minimum degradation as indicated by the absence of free GFP, are selected for a secondary screen. These constructs are scaled and a total membrane fraction prepared and solubilized in four different detergents. Following ultracentrifugation to remove detergent-insoluble material, lysates are analyzed by fluorescence detection size exclusion chromatography (FSEC). Monitoring the size exclusion profile by GFP fluorescence provides information about the mono-dispersity and integrity of the membrane proteins in different detergents. Protein: detergent combinations that elute with a symmetrical peak with little or no free GFP and minimum aggregation are candidates for subsequent purification. Using the above methodology, the heterologous expression in *E. coli *of SED (shape, elongation, division, and sporulation) proteins from 47 different species of bacteria was analyzed. These proteins typically have ten transmembrane domains and are essential for cell division. The results show that the production of the SEDs orthologues in *E. coli *was highly variable with respect to the expression levels and integrity of the GFP fusion proteins. The experiment identified a subset for further investigation.

**Figure:**
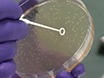


## Introduction

It has been estimated that membrane proteins account for approximately 20-30% of the genes coded in all sequenced genomes ^1^, including the human genome ^2^. Given their critical role in many biological processes, for example transport of metabolites, energy generation and as drug targets, there is intense interest in determining their three dimensional structure. However compared to soluble proteins, membrane proteins are highly under-represented in the Protein Data Bank. In fact, of the 99,624 released structures in the PDB (http://www.rcsb.org/pdb/) only 471 (http://blanco.biomol.uci.edu/mpstruc/) are classified as membrane proteins. This reflects the technical difficulties of working with membrane proteins which are naturally expressed at relatively low levels; rhodopsin is one of a few exceptions^3,4^. Over-expression of recombinant versions in heterologous cells often results in misfolding and poor targeting to the membrane which limits the overall level of production. Selecting the best detergent for extraction and solubilization of a membrane protein once expressed makes subsequent purification difficult and requires careful optimization.

*Escherichia coli *remains the most commonly used expression host for membrane proteins accounting for 72% (http://blanco.biomol.uci.edu/mpstruc/) of structures solved to date which are almost exclusively from bacterial sources. Specific *E. coli *strains, C41(DE3) and C43(DE3), have been isolated which do not lead to the over-expression of membrane proteins and hence avoid the effects of toxicity observed for other BL21 strains^5,6^. Tuning the level of recombinant membrane protein expression appears to be critical for optimizing the level of production^6,7^.

In many cases successful production of membrane proteins for structure determination has required the evaluation of multiple versions including amino- and carboxy-terminal deletions, multiple orthologues to exploit natural sequence variation and different fusion proteins^6-8^. Therefore, a method for the expression screening of multiple membrane proteins in parallel is essential. One commonly used approach is to fuse the protein to green fluorescent protein (GFP) enabling expression to be tracked without the need to purify the protein. In this way, information about expression level, stability and behavior in different detergents can be assessed with small amounts of un-purified material^9-12^.

In this article we describe a streamlined protocol for cloning and expression screening of membrane proteins in *E. coli *using fusion to GFP as a reporter of production and subsequent characterization of detergent: protein complexes (**Figure 1**).

## Protocol

### 1. Construction of Expression Vectors

1. Use PCR Infusion cloning to construct up to 96 expression plasmids in parallel using the vectors pOPINE-3C-GFP and pOPIN-N-GFP which add either cleavable C-terminal or N-terminal GFP-His6 fusions to the sequences^13^ respectively. **Note: **The choice of vector is dictated by the topology of the protein since the GFP needs to be expressed in the cytosol, thus for a protein with a cytosolic N-terminus and extracellular C-terminus we would use pOPIN-N-GFP and for an extracellular N-terminus and cytosolic C-terminus in we would use pOPINE-3C-GFP. Where both termini are cytosolic we would use pOPINE-3C-GFP unless there is functional reason not to tag the C-terminus.
2. Amplify the DNA sequences encoding the membrane proteins with primers incorporating the following extensions shown: pOPINE-3C-GFP (Forward primer — AGGAGATATACCATG; Reverse primer — CAGAACTTCCAGTTT) and pOPIN-N-GFP (Forward primer — AAGTTCTGTTTCAGGGCCCG; Reverse primer — ATGGTCTAGAAAGCTTTA).
3. Analyze the PCR reaction by agarose gel electrophoresis. Once clean PCR products have been obtained, add 5 U DpnI to each well (make up in 5 µl of 1x DpnI reaction buffer). Purify the PCR products using magnetic beads in a 96-well format. **Note: **DpnI is added to remove the template vector; this step is not necessary if genomic DNA is used as template.
4. Add 1 μl (100 ng) of the appropriate linearized vector to each well of a PCR plate.
5. Using a multichannel pipettor add x μl of purified PCR insert to the appropriate wells of the PCR plate. Ensure that all products are in the range of 10-250 ng.
6. Add (9-x) μl of water to the well and transfer the whole 10 μl to a tube containing the dry-down in-fusion reagent. Leave for 30 sec.
7. Mix contents briefly by pipetting up and down. Transfer the reactions back to the original PCR plate and seal.
8. Incubate for 30 min at 42 °C in a thermocycler.
9. Immediately transfer the reactions to ice as soon as the reaction is complete and add 40 μl of TE (10 mM Tris, pH 8.0, 1 mM EDTA).
10. Freeze at once or transform into competent cells. For transformation, use 3 µl of the diluted reaction per 50 μl aliquot of high competency (> 5 x 10^9^ transformants/µg) competent *E. coli *cells.
11. Add 1 ml of LB agar supplemented with 50 µg/ml carbenicillin per well of a 24-well tissue culture plate (for cloning, prepare wells for 2 x number of constructs).
12. Following outgrowth, Following outgrowth plate out 25 μl and 25 μl of a 1 in 5 dilution of the culture onto the agar containing 24-well tissue culture plates.
13. Spread the cells by gently tilting the plate.
14. Dry the plate with the lid off for 10-15 min at 37 °C or in a flow hood at room temperature.
15. Replace the lid and turn the plate upside down and incubate overnight at 37 °C.
16. Pick two colonies and mini-prep the vectors.
17. PCR verify the constructs using the construct specific reverse primer and a vector specific forward primer (*e.g.* pOPIN_FOR GAC CGA AAT TAA TAC GAC TCA CTA TAG GG).

### 2. Transformation of *E. coli E*xpression Strains

**Note:** Prior to performing this protocol, *E. coli *competent cells are made, aliquotted and stored frozen at -80 °C as described previously^13^. Membrane protein expression is routinely screened in two strains, Lemo21(DE3) and C41(DE3)pLysS. To aid interpretation of the results it can be helpful to include a positive control, *e.g.* a plasmid expressing GFP or a membrane protein fused to GFP known to express and an empty vector negative control.

1. Thaw the aliquotted *E. coli *on ice. Add 3 µl of mini-prepped expression plasmid to each aliquot of competent cells.
2. Incubate the mix on ice for 30 min.
3. Heat-shock the cells for 30 sec at 42 °C.
4. Allow cells to recover on ice for 2 min and then add 300 µl of power broth to each tube.
5. Incubate for 1 hr in a 37 °C static incubator.
6. Prepare the LB Agar in a 24-well tissue culture plate, supplemented with 50 µg/ml carbenicillin and 35 µg/ml chloramphenicol. Add rhamnose to a final concentration of 0.25 mM to the wells containing Lemo21 (DE3). **Note: **If the product is likely to be toxic the wells containing C41(DE3) pLysS can be supplemented with 1% (w/v) glucose.
7. Transfer 30 µl of cells from the transformation mix onto the LB Agar plates. Spread and dry the plate as described in 1.13-1.15.

### 3. Preparation of Overnight Starter Cultures

**Note: **Always prepare overnight cultures the day after transformation never from an old transformation plate.

1. Add 0.7 ml power broth to each well of a 96-well deep-well plate supplemented with 50 µg/ml carbenicillin and 35 µg/ml chloramphenicol. Add rhamnose to the wells containing Lemo21 (DE3) to a final concentration of 0.25 mM. Optionally, supplement C41(DE3) pLysS with 1% (w/v) glucose; see above (2.6 note).
2. Pick one colony from the overnight transformation plates into each well. Seal the block with a gas-permeable seal.
3. Shake the block at 250 rpm in an incubator with a large throw (*e.g.* a 25 mm orbit) or at 600 rpm in an incubator with a small throw (*e.g.* a 4.3 mm orbit) at 37 °C overnight.

### 4. Growth and Induction of Cultures

1. Add 3 ml of power broth supplemented with 50 µg/ml carbenicillin and 35 µg/ml chloramphenicol to each well of 24-well deep-well plate. Add L-rhamnose (and glucose) as described in section 3.1. Use 3 ml of culture to allow harvesting in duplicate and to allow for some evaporation of water through the gas permeable seal.
2. Set up the expression cultures by adding 150 µl of Lemo21 (DE3) or C41(DE3) pLysS overnight cultures to each well of the 24-well deep-well blocks.
3. Shake the blocks at 37 °C until the average O.D. at 595 nm is ~0.5 through the plate.
4. Induce expression by adding IPTG to the cultures to a final concentration of 1.0 mM IPTG.
5. Grow the cultures overnight (18-20 hr) with shaking at 20 °C.

### 5. Analysis of Expression by In-gel Fluorescence

1. Harvest in duplicate. Transfer 1 ml of culture from each well into a square well, conical bottom, 96-well deep-well block. **Note: **Harvest in duplicate in case of breakage of the block or to allow the testing of an alternative detergent if desired.
2. Seal the blocks and harvest the cells by centrifugation at 6,000 x g for 10 min.
3. Invert the block and decant the media. Tap the block gently onto a paper towel to drain remaining media.
4. Seal the plates and store at -80 °C for a minimum of 20 min. Optionally, leave the experiment at this point and the block processed when convenient.
5. De-frost the pellets for 20 min at room temperature.
6. Use either a multi-channel pipette or an orbital shaker (1,000 rpm for 10 min) at 4 °C to resuspend the cells completely in 200 µl of lysis buffer (50 mM NaH_2_PO_4_, 300 mM NaCl 10 mM Imidazole 1% v/v Tween 20, adjust the pH to 8.0 using NaOH).
7. Add 20 µl of the DLPI solution (20 µl DNaseI (4,000 units/ml), 20 mg lysozyme, and 20 µl protease inhibitor cocktail in a final volume of 2 ml of water). Incubate for 10 min with shaking (~1,000 rpm) at 4 °C.
8. Add 25 µl of 10% *n*-Dodecyl β-D-maltoside (DDM).
9. Incubate for 60 min with shaking (~1,000 rpm) at 4 °C.
10. Centrifuge the deep-well block at 6,000 x g for 30 min at 4 °C.
11. Transfer 10 μl of the cleared lysate to a microtitre plate and add 10 μl of SDS PAGE gel loading buffer (100 mM Tris, pH 6.8, 4% w/v SDS, 0.2% w/v Bromophenol blue, 10% v/v β-mercaptoethanol, and 20% v/v glycerol). **CAUTION! DO NOT BOIL THE SAMPLE**.
12. Load 10 μl of sample from step 5.11/well onto SDS PAGE gel(s) and run at 100-120 V (constant voltage) in a cold room until the dye front reaches the bottom (2-2.5 hr).
13. Place the gel onto an imager (*e.g.* with a blue-light filter to detect the GFP fusion proteins). The exposure time is chosen to ensure that the brightest bands are not saturated.

### 6. Scale-up of Cell Cultures

1. Transform an aliquot of competent *E. coli *as described in section 2.
2. Pick a colony into 10 ml of power broth (supplemented as described in section 3) and grow overnight at 37 °C.
3. Dilute 5 ml of the overnight culture into 500 ml power broth.
4. Grow with shaking at 250 rpm at 37 °C until the O.D. at 595 nm is ~0.5.
5. Induce expression by adding IPTG to a final concentration of 1.0 mM.
6. Grow overnight with shaking at 250 rpm at 20 °C.
7. Harvest cells by centrifugation at 5,000 x g for 15 min.
8. Discard supernatant. Cell pellets can be stored at -80 °C.

### 7. Preparation of Membranes

1. Thaw cells (if necessary) at room temperature and resuspend in 1x PBS pH 7.5 at a volume of 5ml per gram of cell pellet; Increase volume as necessary until viscosity reduces to a runny consistency. Add MgCl_2_ to final concentration of 1 mM, DNAse I to final concentration of 10 μg/ml and one pre-packaged protease inhibitor tablet per 20 g of cell pellet. Break open cells using a chilled cell disrupter at a pressure of 30 kpsi.
2. Pellet the unbroken cells and debris at 30,000 g for 15 min at 4 °C. Transfer supernatant to an ultra-centrifugation tube.
3. Collect the total membrane fraction by spinning down the supernatant in an ultra-centrifuge at 200,000 x g for 1 hr at 4 °C.
4. Discard the supernatant.
5. Re-suspend the membrane pellet in ice-cold 10 ml PBS pH 7.5 using a cell homogenizer. 1 ml resuspended membranes are required for each detergent. Optionally, at this stage, flash freeze the membrane suspension in liquid nitrogen and store at -80 °C.

### 8. Solubilization and Analysis of the Membrane Fraction

1. Thaw membrane fraction (if necessary), and for each detergent to be used for solubilization, aliquot 900 µl of the membrane suspension into a 1.5 ml polyallomer micro-centrifuge tube.
2. Add 100 µl of each freshly prepared detergent (10% w/v solutions of DDM, DM, Cymal-6 and LDAO) to a final concentration of 1% to the specified 1.5 ml tube. For solubilization of eukaryotic membrane proteins cholesterol hemisuccinate (0.2% final concentration) can be added to the detergents.
3. Incubate the mixtures at 4 °C for 1 hr with mild agitation.
4. Pellet the detergent insoluble fraction with a bench top ultra-centrifuge, ensuring tubes are at least half-full, at 150,000 g at 4 °C for 45 min and retain supernatant. **Note:** The effectiveness of detergent solubilization can be estimated using a fluorescent plate reader by measuring the GFP fluorescence of the solubilized membranes with the detergent insoluble fraction resuspended in the same volume of PBS.
5. Analyze the mono-dispersity of the different detergent : membrane protein complexes using fluorescence-detected size exclusion chromatography (FSEC).
6. For FSEC analysis, equilibrate a size exclusion column with a broad fractionation range, *e.g.* 5,000 to 5,000,000 in molecular weight, with running buffer (20 mM Tris pH 7.5, 150 mM NaCl, 0.03% DDM) at a flow rate of 0.3 ml/min. Use this buffer for all samples*, i.e.* it is not changed for each detergent tested.
7. Inject 100 µl of solubilized membranes onto the column at a flow rate of 0.3 ml/min. Monitor elution profile using fluorescence optics (excitation at 488 nm and emission at 512 nm). If an inline fluorescence monitor is not available, collect the fractions and read the fluorescence in a plate reader.
8. Import elution volume and fluorescence intensity data into a spreadsheet program for graphical display.
9. Analyse remaining solubilized membrane material by in-gel fluorescence as described in section 5.

## Representative Results

The SEDS family of proteins (shape, elongation, division, and sporulation) is essential for bacterial cell division. These integral proteins typically have ten transmembrane domains with both amino and carboxy termini inside the cell. To exemplify the protocol described above, 47 SEDs orthologues were cloned into the vector pOPINE-3C-eGFP. A small scale expression screen of the constructs was carried out in two *E .coli *strains, Lemo21(DE3) grown in the presence of 0.25 mM rhamnose to block leaky expression and C41(DE3) pLysS, which are routinely used for the expression of membrane proteins^5-8^.

Detergent solubilized lysates of induced cultures were analyzed by SDS-polyacrylamide electrophoresis. Using in-gel fluorescence to visualize expressed GFP fusion proteins showed that between the two strains 38 out of the 47 SEDs constructs were produced. Interestingly there were differences between the two expression strains. Of the 38 expressed constructs, only 18 were produced in both strains, 14 only expressed in Lemo21(DE3) and 6 only in C41(DE3) pLysS. Overall, there was a higher success rate for the Lemo21(DE3) compared to the C41(DE3) pLysS (38 vs 24). However the observation that some proteins appeared to be strain specific means that screening in both is useful.

Representative data for 24 of the 47 SEDs constructs are shown in **Figure 1**. The intensity of the bands gives an indication of the level of expression, *e.g.* the fusion protein in well C4 in **Figure 1A** and **1B** is expressed well in both strains however the additional lower molecular weight bands suggest that the protein is partially degraded. In many lanes there is a band which corresponds in size to GFP alone. A qualitative assessment of the integrity of the protein can be carried out by looking at the intensity of fusion protein relative to the free GFP; *e.g.* G4 and H4 are completely broken down to free GFP in C41(DE3) pLysS (**Figure 1B**) whereas in the Lemo21(DE3) there is some intact protein (**Figure 1A**). For 14 out of the 38 expressed proteins the intensity of the free GFP band is similar to that of the fusion protein, for 21 the intensity of the free GFP band is greater than that of the fusion protein and a minority of the fusion proteins (3/38 expressed) *e.g.* F6 and G6 in **Figure 1A** have relatively little free GFP compared to the fusion protein.

Two of the constructs that expressed well in the primary screen B5 (high intensity bands for free GFP and fusion proteins) and G6 (little free GFP) were expressed and a total membrane fraction prepared from 500 ml cell culture. Resuspended membranes were divided into aliquots and analyzed by fluorescence-detected size exclusion chromatography (FSEC). The FSEC profiles are presented in **Figure 2A** and **2B** respectively and show that the two SEDs proteins behave differently in the four detergents tested. In one case (**Figure 2A**: sample B5) the protein only gave a symmetrical peak with little aggregation in DDM which would therefore be the detergent of choice for subsequent purification. By contrast the other fusion protein (**Figure 2B**: sample G6) gave a similar profile in all detergents tested.


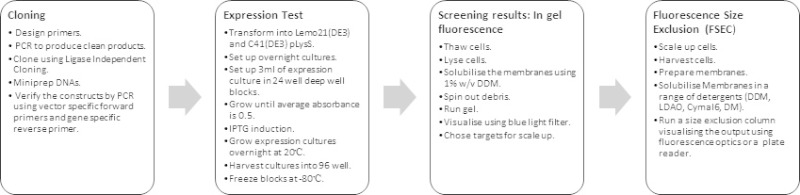
 **Figure 1: Diagram of workflow for screening membrane protein expression in *E. coli *using a GFP reporter.**


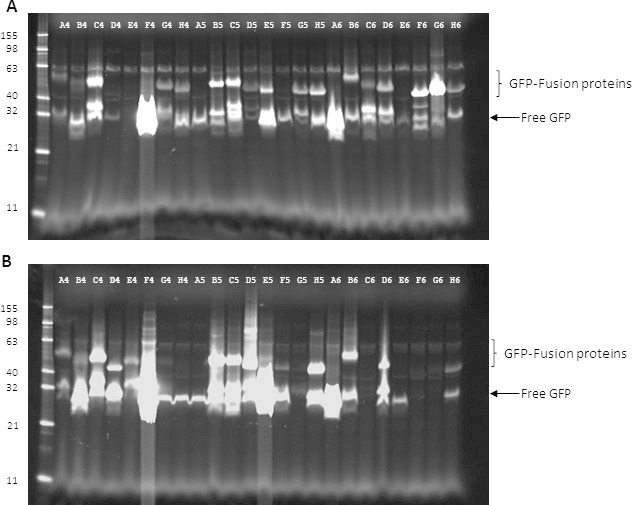
**Figure 2: Primary screen of membrane protein expression using in-gel fluorescence.** Detergent lysates of *E. coli *cells were analyzed by SDS-PAGE and gels imaged using Blue Epi illumination and a 530/28 filter. The results are shown for 24 constructs (labeled A4-H6 based on their position in a 96-well plate). The positions of the bands for free GFP and GFP fusion proteins are indicated. **(A)** Proteins expressed in Lemo21(DE3). **(B)** Proteins expressed in C41(DE3) pLysS.


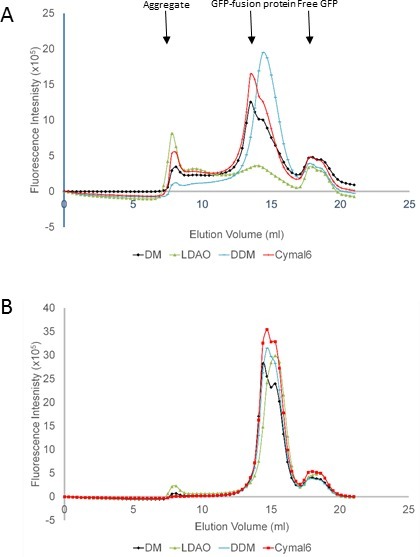
**Figure 3: Secondary screen of membrane protein expression using Fluorescence-detected size exclusion chromatography (FSEC).** Total membrane fractions were extracted in four different detergents and the solubilized membrane proteins analyzed by size exclusion chromatography using a FPLC system coupled to a fluorescence detector. FSEC profiles for **(A) **membrane protein B5 and **(B)** membrane protein G6. The position of the peaks corresponding to aggregated protein, solubilized GFP fusion protein and free GFP are indicated. The detergents tested are indicated below the profiles.

## Discussion

In the protocol described in this article, ligation independent cloning into a GFP reporter vector is combined with fluorescence detection to rapidly screen the relative expression of multiple membrane proteins in *E. coli*. Vectors can be made in four working days with primary expression screening taking a further week. In-gel fluorescence of detergent lysates of whole cells is used to rank expression hits for further analysis in a secondary screen. The primary expression screen as presented does not require any specialist equipment apart from a shaker incubator suitable for growing cells in deep well blocks. All other liquid handling involving 96-well SBS plates can be carried out using multi-channel pipettes. The protocol can be readily modified to include other *E.coli* strains grown under different expression conditions (*e.g.* lower temperature). In the case of the LEMO21(DE3) titrating the level of rhamnose (from 0 to 2.0 mM) added to modulate the rate of transcription can increase the level of expression of some membrane proteins^7^ . Detergents other than DDM could be used for extraction in the primary screen though harsher detergents may solubilize proteins from inclusion bodies and therefore give false positives. Clearly, the more elaborate the initial screen becomes, the more time-consuming the experiment.

The secondary screen involves preparing a total membrane fraction from the expression hits identified in the primary screen. This is a critical step as it will confirm the localisation of the fusion proteins to the membrane of the *E. coli* cells. Subsequent extraction of the GFP fusion protein in different detergents is monitored by fluorescence-detected size exclusion chromatography of solubilized material to assess the mono-dispersity of the detergent-protein complexes. This is another critical step since it identifies the most appropriate detergent for solubilization of the membrane protein for subsequent downstream processing. However, experience shows that further optimization of the choice of detergent(s) may still be required during purification and crystallization. Evidence of uniform behavior in different detergents including ones with a relatively small micelle size (*e.g.* LDAO) appears to be good indicator of a propensity to form well diffracting crystals at least for prokaryotic membrane proteins^14^. In this respect the profile shown for the membrane protein in **Figure 2B** appears highly favorable.

The main advantage of using a GFP reporter is that it gives a rapid read-out of expression in un-purified samples. A disadvantage is that the GFP fusion protein has to be removed during the purification of the protein for crystallization. In the case of the vectors used here, a rhinovirus 3C protease site enables cleavage of the GFP. Alternatively, it is straightforward to re-clone candidate proteins, identified in the screen, into vectors which only add a polyhistidine or other affinity tag for subsequent purification. This assumes that fusion to GFP is not necessary for expression. The main alternative to using fusion to GFP for detection of membrane protein expression and solubilization is to add only a histidine tag. However this approach typically requires starting with a membrane fraction^15^ which for the evaluation of many variants would become very time-consuming.

In summary, the main purpose of the protocol described in this article is to enable a large number of membrane protein sequence variants to be rapidly evaluated in terms of expression and detergent solubilization and prioritized for deeper analysis.

## Disclosures

The authors have nothing to disclose.
